# Fatigue and its correlation with anxiety and depression in patients with multiple sclerosis in China

**DOI:** 10.3389/fneur.2025.1604540

**Published:** 2025-07-07

**Authors:** Han Wang, Rongrong Wang, Runze Zhao, Gaopan Zhang, Guoxun Zhang, Xiongfei Zhao

**Affiliations:** ^1^Department of Neurology, Yan'an University Medical College No. 3 Affiliated Hospital, Xianyang, China; ^2^Yan'an University Medical College, Yan'an, China; ^3^Department of Ophthalmology, Xijing Hospital, Air Force Medical University, Xi'an, China

**Keywords:** multiple sclerosis, epidemiology, fatigue, anxiety, depression

## Abstract

**Background:**

Multiple Sclerosis (MS) is a chronic, progressive, immune-mediated disease of the central nervous system. Fatigue is a common and disabling symptom in patients with MS (PwMS). Some psychological factors, such as depression, stress, and anxiety, also appear to be related to these issues.

**Objectives:**

The study aimed to investigate the fatigue conditions of PwMS in China and its influencing factors, as well as to explore the correlation between fatigue with anxiety and depression.

**Methods:**

This study was a cross-sectional study conducted through the Internet, which collected demographic characteristics, clinical data, Modified Fatigue Impact Scale (MFIS), Beck Anxiety Inventory (BAI), and Beck Depression Inventory-II (BDI-II).

**Results:**

A total of 366 PwMS were included in this study. MFIS showed a median fatigue total score of 40.0 (interquartile range [IQR] 26.0–52.0), with 55.7% (*n* = 204) of patients experiencing fatigue. The scores on MFIS were positively correlated with age, disease duration, BAI score, and BDI-II score.

**Conclusion:**

The prevalence of fatigue among Chinese PwMS is 55.7%. Age, education, employment, marital status, type of disease, and disease duration are all factors influencing the fatigue rate in PwMS. Fatigue in PwMS exhibit strong positive correlations with depression and anxiety.

## 1 Introduction

Multiple Sclerosis (MS) is an immune-mediated disease primarily characterized by inflammatory demyelinating lesions in the central nervous system (CNS), with features of inflammation, demyelination, gliosis, and neuronal loss. The lesions predominantly affect the white matter. The etiology remains unclear and may be associated with a variety of factors including genetics, environment, and viral infections (1). It is estimated that globally, 2.8 million people are affected by MS (35.9 per 100,000 individuals) (2). The incidence rate of MS in China is 0.235 per 100,000 people. MS predominantly affects young and middle-aged individuals (3), with a male-to-female ratio of 1:3 (4).

Patients with multiple sclerosis (PwMS) exhibit a variety of clinical manifestations, including decreased vision, diplopia, sensory and motor disturbances in the limbs, ataxia, fatigue, and bladder or bowel dysfunction. Common psychological dysfunctions in PwMS include depression, anxiety, stress, and sleep disorders (5, 6). As the disease progresses, these symptoms impact the patient's daily life and reduce their quality of life (7, 8).

The pathophysiology of fatigue in PwMS have not been fully elucidated and may involve mechanisms related to immune, metabolic, and inflammatory aspects such as demyelination, impaired cellular metabolism, and alterations in neurotransmitter function (9). Some studies indicated that PwMS who have anxiety disorders are significantly more likely to experience fatigue, pain, and sleep problems, which are exacerbated by the coexistence of depression (10).

The purpose of this study is to understand the prevalence of fatigue in PwMS and its influencing factors, and to explore the correlation of fatigue to anxiety and depression.

## 2 Materials and methods

### 2.1 Study design and participants

This was a cross-sectional study conducted from January 4th to 22nd, 2024, by distributing an online survey questionnaire to PwMS in China. The study was fully compliant with national and international regulations, as well as the Declaration of Helsinki (2013). This study was approved by the Ethics Committee of Yan'an University Medical College No. 3 Affiliated Hospital (number: YDXY-KY-2023-014). All enrolled patients agreed with the participation in the project and the usage of anonymized data.

Inclusion criteria: The study included PwMS from the patient database of Yan'an University Medical College No. 3 Affiliated Hospital. All PwMS were diagnosed according to the 2017 revised McDonald criteria (11), and their diagnosis was confirmed by a neurologist. Participants were aged ≥18 years old. Moreover, they had signed an informed consent form and had the ability to read and correctly understand the content of the scales and provide responses. Exclusion criteria: Patients were excluded if they could not complete the survey questionnaire correctly or had invalid responses. Also excluded were patients with clinically isolated syndrome (CIS) in the disease subtype of MS, as well as those with other neurological immune diseases, such as neuromyelitis optica spectrum disorder (NMOSD) and MOG antibody-positive diseases. Patients with severe cognitive impairment or other functional disabilities that could affect scale assessment were also excluded. In addition, excluded were patients with other diseases that might affect fatigue (such as cancer, severe cardiopulmonary diseases, renal failure, migraine, hypothyroidism, and severe sleep disorders). Finally, patients who had been in the acute phase of MS due to a recent onset or relapse with in the past 4 weeks were excluded.

### 2.2 Questionnaire data collection

An anonymous questionnaire survey was conducted using the “Wenjuanxing” software (https://www.wjx.cn/). The questionnaire is divided into five parts, including demographic characteristics, clinical characteristics, and Modified Fatigue Impact Scale (MFIS) (12), Beck Anxiety Inventory (BAI) (13), Beck Depression Inventory-II (BDI-II) (14). For demographic characteristics, we collected data on gender, age, current residence, education, employment, and marital status. For clinical characteristics, we gathered information on disease type of MS, which is categorized as relapsing-remitting MS (RRMS), primary-progressive MS (PPMS) and secondary-progressive MS (SPMS) (11). We also collected data on disease duration, current medications, use of fampridine sustained-release tablets, use of modafinil or amantadine, history of depression disorders, use of anti-depressant medications, history of anxiety disorders, and use of anti-anxiety medications. For fatigue assessment, the MFIS is used to measure the fatigue condition of patients over the past 4 weeks. It is divided into three subscales: cognitive, physical, and psychosocial, with a total of 21 items, including 10 items on cognitive function, 9 items on physical function, and 2 items on psychosocial function. Each item is scored based on the frequency of symptom occurrence from “none” to “almost always” with scores ranging from 0 to 4. The total score ranges from 0 to 84, with higher scores indicating more severe fatigue and a greater impact on quality of life. An MFIS total score of ≥38 points can be defined as a state of fatigue (12, 15, 16). For anxiety assessment, the BAI is used to measure the 21 cognitive and somatic symptoms of clinical anxiety experienced by patients over the past week. The scale consists of 21 items, with each item scored according to the severity of symptoms from “none” to “severe” ranging from 0 to 3 points. The total score ranges from 0 to 63, with higher scores indicating a more severe level of anxiety. The scoring ranges can be defined as 0–7 (normal), 8–15 (mild anxiety), 16–25 (moderate anxiety), and 26–63 (severe anxiety) (13). For depression assessment, the Beck Depression Inventory-II (BDI-II) is used to measure the severity of depression in patients over the past 2 weeks. The scale consists of 21 items, with each item scored according to the severity of symptoms from “none” to “severe” ranging from 0 to 3 points. The total score ranges from 0 to 63, with higher scores indicating a more severe level of depression (14). The scoring range can be categorized as 0–13 (normal), 14–19 (mild depression), 20–28 (moderate depression), and 29–63 (severe depression^)^ (17).

### 2.3 Statistical analysis

Data analysis was conducted using the SPSS 27.0 statistical software (IBM, USA). Normally distributed quantitative data were presented as mean ± standard deviation (SD), while non-normally distributed quantitative data were presented as median and interquartile range [M (P25, P75)]. Categorical data are expressed as frequency and percentage (%). Chi-square tests and non-parametric Mann–Whitney U tests were used to analyze the factors affecting fatigue in PwMS. Spearman's correlation analysis was utilized to explore the correlations of fatigue to anxiety and depression. In the correlation analysis, the strength and direction of the relationships between variables were quantified using the correlation coefficient (*R*). The *R*-value ranges from −1 to 1, where values closer to 1 or −1 indicate a stronger positive or negative relationship, respectively, and values near 0 suggest little to no correlation. Statistical significance was determined at the *P* < 0.05 level.

## 3 Results

### 3.1 Baseline characteristics of PwMS

**Figure 1:**
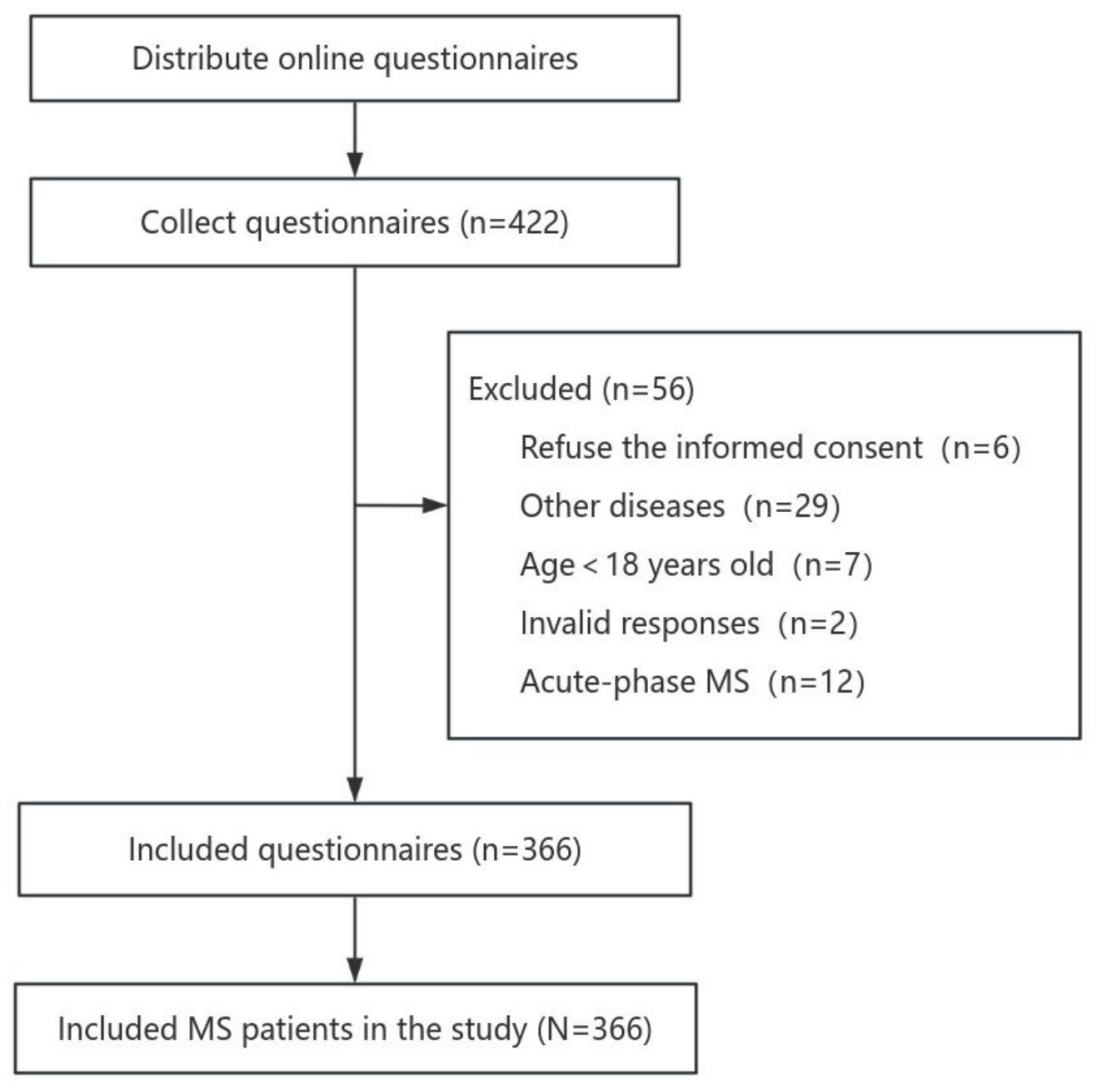
Flow diagram representing the data selection process. MS, multiple sclerosis.

**Figure 2:**
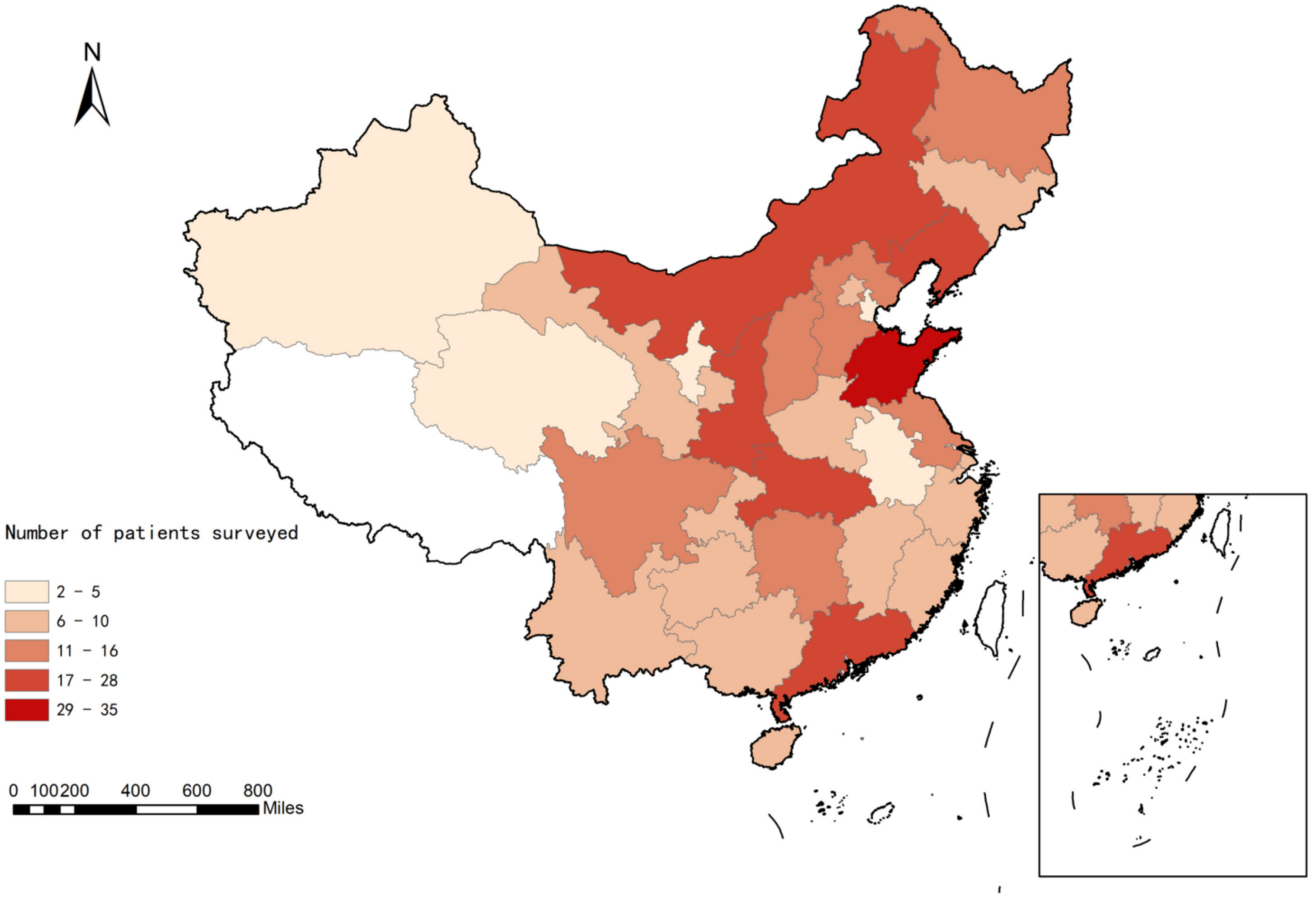
National distribution map of patients participating in this online survey. The intensity of the color reflects the number of participants from each province varies.

A total of 422 questionnaires were collected, with 366 valid questionnaires, yielding a response rate of 86.7%. Exclusions accounted for 56 questionnaires, including those who refused to provide informed consent (*n* = 6), individuals under the age of 18 years old (*n* = 7), those with other diseases (*n* = 29), invalid responses (*n* = 2), and patients with acute-phase MS (*n* = 12) (Figure 1). A total of 366 MS patients were included in this study, and the baseline characteristics are shown in Table 1. Among the patients, 75.4% (*n* = 276) were female and 24.6% (*n* = 90) were male, with a gender ratio of 3:1 (female:male). The median age was 33.5 (28.0, 42.0) years old. 246 (67.2%) patients were graduated from university. In this study, 222 (60.7%) patients were employed, and 115 (31.4%) patients were unemployed. Among the patients, 114 (31.1%) were single, and 227 (62.0%) were married. The national distribution map of the patients' residences in this study was shown in Figure 2, with the highest number of respondents from Shandong Province (35 patients), followed by Guangdong Province (28 patients), Inner Mongolia (27 patients), and Shaanxi Province as well as Hubei Province (both with 26 patients).

**Table 1:** Demographic and clinical data of PwMS investigated in this study (*N* = 366).

**Variables**	**Patients (*n =* 366)**	**Non-fatigue group (*n =* 162)**	**Fatigue group (*n =* 204)**	***P***
**Gender**				
Male	90 (24.6%)	34 (21.0%)	56 (27.5%)	0.154^*^
Female	276 (75.4%)	128 (79.0%)	148 (72.5%)	
**Age (years)**	**33.5 (28.0, 42.0)**	**31.0 (27.0, 36.3)**	**36.0 (30.0, 47.0)**	**< 0.001^‡^**
**Groups of age (years)**				
18 ≤ Age < 28	90 (24.6%)	54 (33.3%)	36 (17.6%)	< 0.001^*^
28 ≤ Age < 38	142 (38.8%)	71 (43.8%)	71 (34.8%)	
38 ≤ Age < 48	77 (21.0%)	30 (18.5%)	47 (23.0%)	
48 ≤ Age < 58	42 (11.5%)	4 (2.5%)	38 (18.6%)	
Age ≥ 58	15 (4.1%)	3 (1.9%)	12 (5.9%)	
**Education**				
Junior high school and below	58 (15.8%)	17 (10.5%)^a^	41 (20.1%)^a^	< 0.001^*^
Senior high school	62 (16.9%)	17 (10.5%)^a^	45 (22.1%)^a^	
University and above	246 (67.2%)	128 (79.0%)^b^	118 (57.8%)^b^	
**Employment**				
Student	29 (7.9%)	16 (9.9%)^a^	13 (6.4%)^a^	< 0.001^*^
Employed	222 (60.7%)	115 (71.0%)^a^	107 (52.5%)^a^	
Unemployed	115 (31.4%)	31 (19.1%)^b^	84 (41.2%)^b^	
**Marital status**				
Single	114 (31.1%)	62 (38.3%)^a^	52 (25.5%)^a^	0.007^*^
Married	227 (62.0%)	95 (58.6%)^a, b^	132 (64.7%)^a, b^	
Divorced	22 (6.0%)	5 (3.1%)^b^	17 (8.3%)^b^	
Widowed	3 (0.8%)	0 (0.0%)^a, b^	3 (1.5%)^a, b^	
**MS subtype**				
RRMS	319 (87.2%)	156 (96.3%)^a^	163 (79.9%)^a^	< 0.001^*^
SPMS	34 (9.3%)	3 (1.9%)^b^	31 (15.2%)^b^	
PPMS	13 (3.6%)	3 (1.9%)^a, b^	10 (4.9%)^a, b^	
**Disease Duration (months)**	**45.0 (17.0, 96.0)**	**31.5 (9.8, 65.3)**	**59.5 (24.0, 133.8)**	**< 0.001^‡^**
**Current medications for MS**				
Teriflunomide	69 (18.9%)	35 (21.6%)	34 (16.7%)	0.745^*^
Dimethyl Fumarate	39 (10.7%)	18 (11.1%)	21 (10.3%)	
Siponimod	64 (17.5%)	26 (16.0%)	38 (18.6%)	
Fingolimod	7 (1.9%)	3 (1.9%)	4 (2.0%)	
Ozanimab	3 (0.8%)	1 (0.6%)	2 (1.0%)	
Ofatumumab	78 (21.3%)	32 (19.8%)	46 (22.5%)	
Rituximab	5 (1.4%)	4 (2.5%)	1 (0.5%)	
Glucocorticoid	15 (4.1%)	4 (2.5%)	11 (5.4%)	
Traditional Immunosuppressants (Azathioprine, Tacrolimus, Cyclophosphamide, Mycophenolate Mofetil, ...)	10 (2.7%)	5 (3.1%)	5 (2.5%)	
Glucocorticoid+ Traditional Immunosuppressants	8 (2.2%)	3 (1.9%)	5 (2.5%)	
Traditional Chinese Medicine Treatment	14 (3.8%)	8 (4.9%)	6 (2.9%)	
None	54 (14.8%)	23 (14.2%)	31 (15.2%)	
**Use of fampridine**				
Yes	80 (21.9%)	11 (6.8%)	69 (33.8%)	< 0.001^*^
No	286 (78.1%)	151 (93.2%)	135 (66.2%)	
**Use of modafinil or amantadine**				
Yes	21 (5.7%)	5 (3.1%)	16 (7.8%)	0.052^*^
No	345 (94.3%)	157 (96.9%)	188 (92.2%)	

This table reflects the demographic characteristics and clinical data of the patients surveyed in this study, as well as a comparative summary between the fatigue group and the non-fatigue group of MS patients. Non-normally distributed quantitative data were presented as median and interquartile range [M (P25, P75)].

MS, multiple sclerosis; PwMS, patients with multiple sclerosis; RRMS, relapsing-remitting multiple sclerosis; SPMS, secondary-progressive multiple sclerosis; PPMS, primary-progressive multiple sclerosis. *P*, *P*-value, indicating the compatibility of observed data with the null hypothesis in significance tests.

* Chi-square test;

‡ Non-parametric test Mann–Whitney *U*-test. ^a, b^Represent the results of pairwise comparisons. If the symbols are the same, it indicates no difference between groups; if the symbols are different, it indicates a difference between groups.

Most of the patient were RRMS, accounting for 87.2% (*n* = 319) of the overall patients, followed by SPMS patients at 9.3% (*n* = 34), and PPMS patients at 3.6% (*n* = 13). The median disease duration was 45.0 (17.0, 96.0) months. The disease modifying therapy (DMT) currently used were shown in Table 1, with ofatumumab accounting for the largest proportion at 21.3% (*n* = 78), followed by teriflunomide at 18.9% (*n* = 69), and siponimod at 17.5% (*n* = 64). 80 (21.9%) patients had a history of taking fampridine sustained-release tablets, and 21 (5.7%) patients had a history of taking modafinil or amantadine (Table 1).

### 3.2 Prevalence of fatigue, anxiety, and depression in PwMS

The survey indicated that 13.9% (*n* = 51) of the patients had a history of depression disorders, with 10.9% (*n* = 40) patients having used anti-depressant medications. And it also shows that 16.1% (*n* = 59) had a history of anxiety disorders, with 10.9% (*n* = 40) patients having used anti-anxiety medications.

The results from the MFIS showed that the median total fatigue score was 40.0 (26.0, 52.0), with a median physical score of 18.5 (11.0, 26.0), a median cognitive score of 18.0 (12.0, 23.0), and a median psychosocial score of 3.0 (1.0, 5.0). The proportion of patients experiencing fatigue (MFIS total score ≥38) was 55.7% (*n* = 204). The results from the BAI indicated that the median total anxiety score was 8.0 (3.0, 16.0), with 172 patients (47.0%) having no anxiety, and 194 patients (53.0%) experiencing anxiety. The results from the BDI-II showed that the median total depression score was 13.0 (6.0, 23.0), with 193 patients (52.7%) having no depressive symptoms, and 173 patients (47.3%) exhibiting depressive symptoms. Significantly, the BAI indicated that 8.7% (*n* = 32) of patients had symptoms of severe anxiety, and the BDI-II showed that 15.8% (*n* = 58) of patients had symptoms of severe depression (Table 2).

**Table 2:** Prevalence of fatigue, anxiety and depression in PwMS in This Study (*N* = 366).

**Variables**	**Patients (*n =* 366)**	**Non-fatigue group (*n =* 162)**	**Fatigue group (*n =* 204)**	***P***
**History of depression disorders**				
Yes	51 (13.9%)	9 (5.6%)	42 (20.6%)	<0.001^*^
No	315 (86.1%)	153 (94.4%)	162 (79.4%)	
**Use of anti-depressant medications**				
Yes	40 (10.9%)	8 (4.9%)	32 (15.7%)	0.001^*^
No	326 (89.1%)	154 (95.1%)	172 (84.3%)	
**History of anxiety disorders**				
Yes	59 (16.1%)	10 (6.2%)	49 (24.0%)	<0.001^*^
No	307 (83.9%)	152 (93.8%)	155 (76.0%)	
**Use of anti-anxiety medications**				
Yes	40 (10.9%)	3 (1.9%)	37 (18.1%)	<0.001^*^
No	326 (89.1%)	159 (98.1%)	167 (81.9%)	
**Fatigue**				
MFIS total score ≥38 points	204 (55.7%)	0 (0.0%)	204 (100%)
MFIS total score	40.0 (26.0, 52.0)	24.0 (17.0, 31.0)	50.5 (44.0, 58.0)	<0.001^‡^
Physical score	18.5 (11.0, 26.0)	10.5 (7.0, 14.0)	25.0 (20.0, 29.0)	<0.001^‡^
Cognitive score	18.0 (12.0, 23.0)	11.0 (7.0, 15.0)	23.0 (18.0, 26.0)	<0.001^‡^
Psychosocial score	3.0 (1.0, 5.0)	1.0 (0.0, 2.0)	5.0 (3.3, 7.0)	<0.001^‡^
**BDI-II Total Score**	**13.0 (6.0, 23.0)**	**6.0 (3.0, 12.0)**	**20.0 (12.0, 29.0)**	**<0.001^‡^**
**Groups of depression**				
Normal (0–13 points)	193 (52.7%)	134 (82.7%)	59 (28.9%)	<0.001^*^
Mild depression (14–19 points)	50 (13.7%)	16 (9.9%)	34 (16.7%)	
Moderate depression (20–28 points)	65 (17.8%)	8 (4.9%)	57 (27.9%)	
Severe depression (29–63 points)	58 (15.8%)	4 (2.5%)	54 (26.5%)	
**BAI total score**	**8.0 (3.0, 16.0)**	**4.0 (1.0, 7.3)**	**14.0 (8.0, 21.0)**	**<0.001^‡^**
**Groups of anxiety**				
Normal (0–7 points)	172 (47.0%)	122 (75.3%)	50 (24.5%)	<0.001^*^
Mild Anxiety (8–15 points)	101 (27.6%)	34 (21.0%)	67 (32.8%)	
Moderate anxiety (16–25 points)	61 (16.7%)	5 (3.1%)	56 (27.5%)	
Severe anxiety (26–63 points)	32 (8.7%)	1 (0.6%)	31 (15.2%)	

Non-normally distributed quantitative data were presented as median and interquartile range [M (P25, P75)].

MFIS, Modified Fatigue Impact Scale; BDI-II, Beck Depression Inventory-II; BAI, Beck Anxiety Inventory.

* Chi-square test;

‡ Non-parametric test Mann–Whitney *U*-test.

### 3.3 Univariate analyses of factors affecting fatigue in PwMS

Patients were divided into fatigue (MFIS total score ≥38 points, *n* = 204) and non-fatigue group (MFIS total score <38 points, *n* = 162), as shown in Table 1. The median age of patients with fatigue was older than that of patients without fatigue (36.0 [30.0, 47.0] vs. 31.0 [27.0, 36.3], *P* < 0.001). There was a significant difference in patients with different education background (*P* < 0.001), with patients having a university degree or above experiencing a lower rate of fatigue compared to those with a high school, middle school, or lower education level. There was a statistically significant difference in the occurrence of fatigue among patients with different occupations (*P* < 0.001) and unemployed individuals had a higher rate of fatigue. The pairwise comparisons revealed that unmarried patients had a lower rate of fatigue than divorced patients. There was a significant difference in fatigue rates among different disease types (*P* < 0.001). SPMS patients showed a markedly high rate of fatigue compared to RRMS patients. The median disease duration was greater in the patients with fatigue than in the patients without fatigue (59.5 [24.0, 133.8] months vs. 31.5 [9.8, 65.3] months, *P* < 0.001). The use of fampridine sustained-release tablets showed a statistically significant difference between the MS fatigue group and the non-fatigue group (*P* < 0.001).

Patients with a history of depression and the use of antidepressant medication, as well as those with a history of anxiety disorders and the use of anti-anxiety medication, all had a significant impact on the incidence of fatigue (*P* < 0.001). The median BAI and BDI-II scores were high in the fatigue group compared to the non-fatigue group (*P* < 0.001). There was a significant difference in the rate of fatigue among patients with no anxiety symptoms, mild anxiety, and moderate to severe anxiety (*P* < 0.001). Similarly, there was a significant difference in the presence of fatigue among patients with no depressive symptoms, mild to moderate depression, and severe depression (*P* < 0.001). There was no significant difference in the rate of fatigue between male and female patients (*P* = 0.154), and the current treatment medication had no differential effect on the rate of fatigue (*P* = 0.745).

The results indicate that age, education, employment, marital status, disease type, and disease duration are all factors influencing fatigue. Gender and the current use of disease-modifying therapies for MS have no significant impact on the presence of fatigue.

### 3.4 Correlation analysis between PwMS baseline characteristics and MFIS scale scores

As shown in Table 3, the fatigue MFIS scale scores (including total scale score, physical score, cognitive score, and psychosocial score) were positively correlated with age, disease duration, BAI scores, and BDI-II scores (all with *R* > 0, *P* < 0.001), and negatively correlated with the education (with *R* < 0, *P* < 0.001).

**Table 3:** Correlation analysis of baseline characteristics and MFIS scale scores of PwMS investigated in this study (*N* = 366).

**Variables**	**Values**	**MFIS Total score**	**Physical score**	**Cognitive score**	**Psychosocial score**
Age (years)	*R*	0.30	0.34	0.15	0.34
	*P*	<0.001	<0.001	0.004	<0.001
Groups of age (years)	*R*	0.31	0.34	0.16	0.34
	*P*	<0.001	<0.001	0.002	<0.001
Education level	*R*	−0.26	−0.24	−0.21	−0.23
	*P*	<0.001	<0.001	<0.001	<0.001
Disease duration (months)	*R*	0.28	0.31	0.14	0.26
	*P*	<0.001	<0.001	0.007	<0.001
BDI-II total score	*R*	0.68	0.58	0.66	0.53
	*P*	<0.001	<0.001	<0.001	<0.001
Groups of depression	*R*	0.63	0.54	0.59	0.48
	*P*	<0.001	<0.001	<0.001	<0.001
BAI total score	*R*	0.64	0.59	0.56	0.51
	*P*	<0.001	<0.001	<0.001	<0.001
Groups of anxiety	*R*	0.59	0.56	0.50	0.47
	*P*	<0.001	<0.001	<0.001	<0.001

This table represents Spearman's correlation analysis. correlation coefficient (R): In the correlation analysis, the strength and direction of the relationships between variables were quantified using the correlation coefficient (R). The *R*-value ranges from −1 to 1, where values closer to 1 or −1 indicate a stronger positive or negative relationship, respectively, and values near 0 suggest little to no correlation.

Fatigue was associated with the older age, the longer disease duration, the higher BAI scores, the higher BDI-II scores, and the lower levels of education.

## 4 Discussion

Based on the distribution map across China in this study, there appears to be a trend of higher incidence rates of MS patients in the northern regions compared to the southern regions. Additionally, we observed that fewer patients from the western regions participated in this survey compared to those from the eastern regions. Studies indicate that MS is showing an increasing global prevalence trend, with the highest rates in North America, Western Europe, and Oceania (>100 cases per 100,000 people), and the lowest rates in countries around the equator (<30 cases per 100,000 people) (18, 19). The geographical distribution of MS incidence in China shows a latitudinal gradient from north to south and an altitudinal gradient from east to west, with residents in high latitude and high altitude areas being more susceptible to MS (20). The differences may be associated with selection biases in the population that completed the survey questionnaires. Other possible reasons include the fact that regions with higher socioeconomic status have better medical conditions in terms of diagnostic technology, infrastructure, and access to specialized physicians, and that some areas with lower economic status may have lower diagnosis rates for MS or later diagnosis timing (21).

Fatigue is one of the most common symptoms associated with MS, with a prevalence ranging from 36.5% to 78.0% (22). Study indicates that fatigue associated with MS is related to the dysfunction and atrophy of gray and white matter, and activity-dependent conduction block in the corticospinal tracts or non-motor pathways may be an important mechanism for fatigue (23). Significant disruptions in cortical activation and inhibitory networks are also associated with the patient's symptoms of fatigue (24). Our study indicates that the prevalence of fatigue among PwMS in this study was 55.7%. Other studies have also reported similar findings. According to the study by Eizaguirre et al., the prevalence of fatigue in MS patients was 51.6% (25). Chalah et al. detected fatigue in 55% of MS patients (26). However, Alsharif et al. found fatigue in 37% of MS patients (27). Rzepka et al. found a fatigue prevalence rate of 42% among RRMS (28). In numerous studies, the prevalence of fatigue in PwMS was even higher, such as a fatigue rate of 73% (29) and 76.4% (30). Javalkar et al. reported that at least 83% of PwMS experience fatigue (31). The differences in fatigue rates were quite large, and the reasons may include: Firstly, the races, countries, and ethnicities of the study subjects differ among various studies. Secondly, the sample sizes of patients included in different studies vary, with larger sample sizes generally offering more reliable fatigue rate statistics than smaller ones. Thirdly, the different inclusion and exclusion criteria in the studies also have a certain impact on the fatigue rate statistics. Fourthly, the fatigue research scales used in the studies are diverse, and the efficacy of the scales and their criteria for classification may also be reasons for the differences observed.

The results of this study show that age, education, occupation, marital status, type of disease, and disease duration are all factors that affect the fatigue rate in PwMS. Gender and the current use of disease-modifying therapies for MS have no significant impact on fatigue. Maier et al. found that in PwMS, the MFIS scores were positively correlated with age, total number of relapses, total disease duration, disability status, and BDI-II scores, and negatively correlated with cognitive performance. And gender is a significant factor affecting fatigue rates, with female MS patients experiencing more fatigue than their male counterparts (32). Similarly, Broch et al. have also shown that women have a higher prevalence of fatigue than men (5). Some studies suggested that factors such as hormonal levels, immune system differences, and taking on more family responsibilities in women could impose physical and emotional burdens, potentially leading to fatigue (4, 33). However, our study demonstrated no significant difference in fatigue rates between men and women (*P* = 0.154). Some studies have also found similar results; Trojan et al. discovered no statistically significant difference in fatigue levels between male and female PwMS (34). Rzepka et al. stated that there is no difference in the incidence and level of fatigue between genders in PwMS (28). The possible reasons for this contradiction are as follows: Although there are biological differences, social role differences, and lifestyle habit differences among different genders, the impact of these factors also varies from individual to individual. Some studies have found that women show greater resilience when facing fatigue, which may reduce its impact on them (35). It should be noted that whether there is a difference in the impact of gender on fatigue rates remains controversial, and further research is needed for explanation.

This study indicates that the fatigue rate among patients with SPMS is significantly higher than among those with RRMS. Similarly, Maier et al. found in their research that patients with RRMS had significantly lower levels of fatigue compared to those with SPMS (*P* = 0.001) (32). However, another study reported no statistically significant difference in the severity of fatigue between RRMS and SPMS patients (34). The reasons for the different outcomes are primarily related to the characteristics of the disease subtypes. Patients with SPMS experience a slowly progressive worsening of disease severity (36), unpredictable disease course, and a high risk of developing severe disability, which may lead to increasingly severe physiological and psychological conditions. Compared to those with RRMS, SPMS patients are older and have a higher degree of disability (32). These factors could contribute to the increased fatigue rates in SPMS patients. Our study found that the MFIS scores were negatively correlated with education level, indicating that lower education levels are associated with more severe fatigue. The reasons for this outcome may include varying levels of disease awareness and different extents of psychological, economic, and social burdens associated with different education levels. This also suggests that after researchers have excluded many demographic and immutable factors affecting fatigue rates, it is necessary to identify, adjust, and control modifiable factors in future clinical work to improve or reduce the fatigue levels in PwMS.

Numerous medications have been used to treat fatigue, including amantadine, modafinil, methylphenidate, and levocarnitine. Amantadinecan influence fatigue by increasing the release of dopamine (37). Harirchian et al. have indicated that both modafinil and levocarnitine have significant effects on fatigue in PwMS (38). Our study found that among patients with fatigue, 16 patients (7.8%) had a history of taking modafinil or amantadine. This suggests two aspects to the researchers: on the one hand, PwMS may not pay attention to their own fatigue symptoms. On the other hand, clinicians should conduct a comprehensive symptom interview and relevant scale assessments during the diagnosis and treatment process of MS, and provide patients with disease knowledge popularization and medication guidance. Fatigue is also influenced by the disease's progression, being more severe during exacerbations in MS. It can also worsen with excessive physical exertion and heat exposure (39, 40). In addition to pharmacological treatments, fatigue symptoms can be alleviated through regular exercise, sleep regulation, psychological interventions, stress management, and heat management strategies (40).

Mobility disorders are among the most common and severely impacting symptoms on the quality of life for PwMS. Approximately 50% of patients require assistance with walking within 15 years of disease onset, and 50% will need to use a wheelchair within 25 years after diagnosis (41, 42). Fampridine sustained-release tablets are a type of potassium (K^+^) channel blocker that can improve walking function (43). Clinical studies have indicated that fampridine sustained-release tablets also have significant improvement effects on other symptoms in adult PwMS, such as fatigue, depression, quality of life, vision, and cognitive function (44). In our study, it was shown that 80 PwMS (21.9%) had a history of taking fampridine sustained-release tablets, among whom 33.8% (*n* = 69) patients experienced fatigue symptoms, and the difference in the use of this medication between the MS fatigue group and the non-fatigue group in PwMS was statistically significant (*P* < 0.001). The interpretation of this result is as follows: The study is a statistical analysis of the history of extended-release amantadine use, it only indicates that patients with fatigue symptoms are more likely to choose this medication, and does not imply that patients using fampridine sustained-release tablets have worse fatigue scores.

The incidence of depression in PwMS is three times higher than in the general population, with 30%−45% PwMS experiencing severe depression (31). The etiology of depression includes immune-inflammatory, immune-genetic, psychological, and specific brain damage in MS (45). Anxiety disorders are common symptoms in MS, with an age-standardized prevalence rate as high as 35.6%. In contrast, the general population has a rate of 29.6% (46, 47). The baseline characteristics survey of our study showed that patients with a history of anxiety accounted for 16.1%, and those with a history of depression accounted for 13.9%. Standard scale assessments revealed that 53.0% of PwMS had anxiety symptoms, and 47.3% had depressive symptoms. These results suggest that PwMS experience a wide range of anxiety and depressive symptoms, and most patients may not seek medical attention and medication in a timely manner. This study results also indicate a strong positive correlation between fatigue with depression and anxiety. Similarly, Thomas et al. found in their study that scores for anxiety and depression are strongly correlated with fatigue indices, and weakly to moderately negatively correlated with quality of life indices (48). Some studies have confirmed the same viewpoint as this study, that MFIS scores for fatigue are positively correlated with BDI-II scores (6, 32, 49). Studies have shown that depressive symptoms and the use of sleeping pills are both significantly correlated with fatigue (50). The possible reasons for the aforementioned study results can be explained as follows: Fatigue and depressive symptoms may cluster together with other symptoms of MS, such as anxiety, sleep problems, or pain, affecting the physical activity behaviors of PwMS. This perspective is based on the concept of symptom clusters and the theory of unpleasant symptoms (51–53). Some studies have also pointed out the shared neurobiological basis for psychological issues in PwMS, for instance, fatigue and depression share common mechanisms, such as psychosocial factors and brain injury (54). Additionally, there is currently a limited literature on the mechanistic explanation for the significant relationship between fatigue and anxiety, with most studies increasingly inclined to view anxiety as a response to an underlying disease, while depression is associated with the anatomically specific sites of lesions (55). There is also literature suggesting that the sympathetic nervous system may play a mediating role between anxiety and fatigue. Anxiety can activate the sympathetic nervous system, leading to increased levels of epinephrine and cortisol in the blood, resulting in a chronic sense of fatigue associated with anxiety, which can be referred to as “adrenal fatigue”. However, systematic research has indicated that there is no evidence to prove that “adrenal fatigue” is an actual existing medical symptom (6, 56). It should be noted that further research is still needed in the future for clinical and research workers to explain the mechanisms behind the occurrence of various symptoms.

The three common comorbidities in MS are depression, fatigue, and anxiety. Studies have shown that lifestyle changes, such as maintaining a lower BMI, engaging in regular physical exercise, and reducing the amount of time spent sitting daily, can alleviate fatigue, anxiety, and depression. These changes play an important role in improving symptoms and quality of life for PwMS (17). Therefore, it is crucial to face emotional disorders head-on, actively engage in self-management, and seek medical help to find the best treatment plan.

This study has several limitations. First, this online questionnaire survey was conducted among PwMS across China, but due to the distribution process relying on online channels, there is an inevitable selection bias in the research subjects. Second, patients' completion of the questionnaire and scales is subjective, which may introduce reporting bias and confounding factors. Additionally, individual differences in physical activity and types of exercise among patients also affect the accuracy of the study. Third, this study is a cross-sectional observational study and is therefore unable to analyze the longitudinal progression of fatigue symptoms, nor can it determine the temporal sequence or causal relationships between variables and fatigue symptoms. As such, the associations identified in this study merely reflect co-occurrence rather than causation. In light of these limitations, we emphasize the need for future research to consider employing longitudinal cohort studies or interventional studies to further explore the temporal relationships and causal pathways between psychological symptoms and fatigue, thereby providing a more robust evidence base for the development of relevant intervention strategies.

## 5 Conclusion

This study investigated the fatigue rate (55.7%) among PwMS in China. Age, education, employment, marital status, disease type, and disease duration are factors that influence the fatigue rate in PwMS. This suggests that controlling these factors could potentially reduce fatigue levels or at least mitigate its adverse effects. The study revealed a strong positive correlation between the overall MFIS and its subdomains' fatigue levels with age, disease duration, depression, and anxiety, and a negative correlation with education. By managing the factors related to MS motor and mental comorbidities, the quality of life for patients can be improved.

## Data Availability

The raw data supporting the conclusions of this article will be made available by the authors, without undue reservation.

## References

[1] Kamm CP Uitdehaag BM Polman CH. Multiple sclerosis: current knowledge and future outlook. Eur Neurol. (2014) 72:132–41. 10.1159/000360528 25095894

[2] Walton C King R Rechtman L Kaye W Leray E Marrie RA. Rising prevalence of multiple sclerosis worldwide: insights from the Atlas of MS, third edition. Mult Scler. (2020) 26:1816–21. 10.1177/1352458520970841 33174475 PMC7720355

[3] Brownlee WJ Hardy TA Fazekas F Miller DH. Diagnosis of multiple sclerosis: progress and challenges. Lancet. (2017) 389:1336–46. 10.1016/S0140-6736(16)30959-X 27889190

[4] Coyle P.K What can we learn from sex differences in MS? J. Pers. Med. (2021) 11:1006. 10.3390/jpm11101006 34683148 PMC8537319

[5] Broch L Simonsen CS Flemmen HØ Berg-Hansen P Skardhamar Å Ormstad H. High prevalence of fatigue in contemporary patients with multiple sclerosis. Mult Scler J Exp Transl Clin. (2021) 7:1838547502. 10.1177/2055217321999826 33796331 PMC7985949

[6] Zekibakhsh Mohammadi N Kianimoghadam AS Mikaeili N Asgharian SS Jafari M Masjedi-Arani A. Sleep disorders and fatigue among patients with MS: the role of depression, stress, and anxiety. Neurol Res Int. (2024) 2024:6776758. 10.1155/2024/6776758 38322749 PMC10843872

[7] Calabresi PA. Diagnosis and management of multiple sclerosis. Am Fam Physician. (2004) 70:1935–44. 15571060

[8] Reich DS Lucchinetti CF Calabresi PA. Multiple Sclerosis. N Engl J Med. (2018) 378:169–80. 10.1056/NEJMra1401483 29320652 PMC6942519

[9] Zimek D Miklusova M Mares J. Overview of the current pathophysiology of fatigue in multiple sclerosis, its diagnosis and treatment options—review article. Neuropsychiatr Dis Treat. (2023) 19:2485–97. 10.2147/NDT.S429862 38029042 PMC10674653

[10] Marrie RA Horwitz RI Cutter G Tyry T Vollmer T. Association between comorbidity and clinical characteristics of MS. Acta Neurol Scand. (2011) 124:135–41. 10.1111/j.1600-0404.2010.01436.x 20880264 PMC3394540

[11] Thompson AJ Banwell BL Barkhof F Carroll WM Coetzee T Comi G. Diagnosis of multiple sclerosis: 2017 revisions of the McDonald criteria. Lancet Neurol. (2018) 17:162–73. 10.1016/S1474-4422(17)30470-2 29275977

[12] Flachenecker P Kümpfel T Kallmann B Gottschalk M Grauer O Rieckmann P. Fatigue in multiple sclerosis: a comparison of different rating scales and correlation to clinical parameters. Multiple Sclerosis J. (2002) 8:523–6. 10.1191/1352458502ms839oa 12474995

[13] Beck AT Epstein N Brown G Steer RA. An inventory for measuring clinical anxiety: psychometric properties. J Consult Clin Psychol. (1988) 56:893–7. 10.1037/0022-006X.56.6.893 3204199

[14] Steer RA Brown GK Beck AT Sanderson WC. Mean Beck Depression Inventory-II scores by severity of major depressive episode. Psychol Rep. (2001) 88:1075–6. 10.2466/pr0.2001.88.3c.1075 11597055

[15] Fisk JD Ritvo PG Ross L Haase DA Marrie TJ Schlech WF. Measuring the functional impact of fatigue: initial validation of the fatigue impact scale. Clin Infect Dis. (1994) 18:S79–83. 10.1093/clinids/18.Supplement_1.S79 8148458

[16] Larson R D. Psychometric properties of the modified fatigue impact scale. Int J MS Care. (2013) 15:15–20. 10.7224/1537-2073.2012-019 24453758 PMC3883028

[17] Rezaeimanesh N Rafiee P Saeedi R Eskandarieh S Sahraian MA Khosravian P. Association of body mass index and physical activity with fatigue, depression, and anxiety among Iranian patients with multiple sclerosis. Front Neurol. (2023) 14:1126215. 10.3389/fneur.2023.1126215 37122312 PMC10134856

[18] Hauser S L Cree B. Treatment of multiple sclerosis: a review. Am J Med. (2020) 133:1380–90. 10.1016/j.amjmed.2020.05.049 32682869 PMC7704606

[19] Global regional and and national burden of multiple sclerosis 1990-2016: a systematic analysis for the Global Burden of Disease Study 2016. Lancet Neurol. (2019) 18:269–85. 10.1016/S1474-4422(18)30443-5 30679040 PMC6372756

[20] Tian DC Zhang C Yuan M Yang X Gu H Li Z. Incidence of multiple sclerosis in China: a nationwide hospital-based study. Lancet Reg Health West Pac. (2020) 1:100010. 10.1016/j.lanwpc.2020.100010 34327341 PMC8315658

[21] Zhang GX Carrillo-Vico A Zhang WT Gao SS Izquierdo Ayuso G. Incidence and prevalence of multiple sclerosis in China and other Asian countries. Neurologí*a (English Edition)*. (2023) 38:159–72. 10.1016/j.nrleng.2020.07.022 37059571

[22] Oliva Ramirez A Keenan A Kalau O Worthington E Cohen L Singh S. Prevalence and burden of multiple sclerosis-related fatigue: a systematic literature review. BMC Neurol. (2021) 21:468. 10.1186/s12883-021-02396-1 34856949 PMC8638268

[23] Vucic S Burke D Kiernan M C. Fatigue in multiple sclerosis: mechanisms and management. Clin Neurophysiol. (2010) 121:809–17. 10.1016/j.clinph.2009.12.013 20100665

[24] Leocani L Colombo B Magnani G Martinelli-Boneschi F Cursi M Rossi P. Fatigue in multiple sclerosis is associated with abnormal cortical activation to voluntary movement—EEG evidence. Neuroimage. (2001) 13:1186–92. 10.1006/nimg.2001.0759 11352624

[25] Eizaguirre MB Ciufia N Roman MS Martínez Canyazo C Alonso R Silva B. Perceived fatigue in multiple sclerosis: the importance of highlighting its impact on quality of life, social network and cognition. Clin Neurol Neurosurg. (2020) 199:106265. 10.1016/j.clineuro.2020.106265 33038658

[26] Chalah MA Kauv P Créange A Hodel J Lefaucheur JP Ayache SS. Neurophysiological, radiological and neuropsychological evaluation of fatigue in multiple sclerosis. Mult Scler Relat Disord. (2019) 28:145–52. 10.1016/j.msard.2018.12.029 30594815

[27] Alsharif ZI Mansuri FA Alamri YA Alkalbi NA Almutairi MM Abu Alkhair AF. The role of exercise on fatigue among patients with multiple sclerosis in the King Fahad Hospital, Madinah, Saudi Arabia: an analytical cross-sectional study. Cureus. (2023) 15:e42061. 10.7759/cureus.42061 37601996 PMC10433400

[28] Rzepka M Toś M Boroń M Gibas K Krzystanek E. Relationship between fatigue and physical activity in a polish cohort of multiple sclerosis patients. Medicina (Kaunas). (2020) 56:726. 10.3390/medicina56120726 33371510 PMC7767485

[29] Ahvenjärvi H Niiranen M Simula S Hämäläinen P Surcel HM Remes AM. Fatigue and health-related quality of life depend on the disability status and clinical course in RRMS. Mult Scler Relat Disord. (2023) 77:104861. 10.1016/j.msard.2023.104861 37442075

[30] Ware M O'Connor P Bub K Backus D McCully K. Investigating relationships among interoceptive awareness, emotional susceptibility, and fatigue in persons with multiple sclerosis. Int J MS Care. (2023) 25:75–81. 10.7224/1537-2073.2022-007 36923579 PMC10010111

[31] Gelfand J M. Multiple sclerosis: diagnosis, differential diagnosis, and clinical presentation. Handb Clin Neurol. (2014) 122:269–90. 10.1016/B978-0-444-52001-2.00011-X 24507522

[32] Maier S Bajko Z Rosescu R. Sociodemographic and clinical determinants of fatigue in multiple sclerosis. Life (Basel). (2023) 13:2132. 10.3390/life13112132 38004272 PMC10672347

[33] Anens E Emtner M Zetterberg L Hellström K. Physical activity in subjects with multiple sclerosis with focus on gender differences: a survey. BMC Neurol. (2014) 14:47. 10.1186/1471-2377-14-47 24612446 PMC3975577

[34] Trojan DA Arnold D Collet JP Shapiro S Bar-Or A Robinson A. Fatigue in multiple sclerosis: association with disease-related, behavioural and psychosocial factors. Mult Scler. (2007) 13:985–95. 10.1177/1352458507077175 17468448

[35] Wylie GR Pra Sisto AJ Genova HM DeLuca J. Fatigue across the lifespan in men and women: state vs. trait. Front Hum Neurosci. (2022) 16:790006. 10.3389/fnhum.2022.790006 35615746 PMC9124897

[36] Brochet B Clavelou P Defer G De Seze J Louapre C Magnin E. Cognitive impairment in secondary progressive multiple sclerosis: effect of disease duration, age, and progressive phenotype. Brain Sci. (2022) 12:183. 10.3390/brainsci12020183 35203948 PMC8870031

[37] Pucci E Branãs P D'Amico R Giuliani G Solari A Taus C. Amantadine for fatigue in multiple sclerosis. Cochrane Database Syst Rev. (2007) 2007:CD2818. 10.1002/14651858.CD002818.pub2 17253480 PMC6991937

[38] Al-Shammari A H Abbood Z A Lateef H F. Assessing the impacts of L-carnitine and modafinil on fatigue in Iraqi multiple sclerosis patients. J Adv Pharm Technol Res. (2023) 14:226–8. 10.4103/JAPTR.JAPTR_225_23 37692018 PMC10483915

[39] Freal J E Kraft G H Coryell J K. Symptomatic fatigue in multiple sclerosis. Arch Phys Med Rehabil. (1984) 65:135–8. 6703889

[40] Kos D Kerckhofs E Nagels G D'hooghe MB Ilsbroukx S. Origin of fatigue in multiple sclerosis: review of the literature. Neurorehabil Neural Repair. (2008) 22:91–100. 10.1177/1545968306298934 17409388

[41] Dendrou C A Fugger L Friese M A. Immunopathology of multiple sclerosis. Nat Rev Immunol. (2015) 15:545–58. 10.1038/nri3871 26250739

[42] Goldenberg MM. Multiple sclerosis review. P T. (2012) 37:175–84. 22605909 PMC3351877

[43] Leussink V I Montalban X Hartung H P. Restoring axonal function with 4-aminopyridine: clinical efficacy in multiple sclerosis and beyond. CNS Drugs. (2018) 32:637–51. 10.1007/s40263-018-0536-2 29992409

[44] Mitsikostas DD Doskas T Gkatzonis S Fakas N Maltezou M Papadopoulos D. A prospective, observational, cohort study to assess the efficacy and safety of prolonged-release fampridine in cognition, fatigue, depression, and quality of life in multiple sclerosis patients: the FAMILY study. Adv Ther. (2021) 38:1536–51. 10.1007/s12325-020-01606-5 33528792 PMC7932964

[45] Masuccio FG Gamberini G Calabrese M Solaro C. Imaging and depression in multiple sclerosis: a historical perspective. Neurol Sci. (2021) 42:835–45. 10.1007/s10072-020-04951-z 33411192

[46] Boeschoten RE Braamse AMJ Beekman ATF Cuijpers P van Oppen P Dekker J. Prevalence of depression and anxiety in Multiple Sclerosis: a systematic review and meta-analysis. J Neurol Sci. (2017) 372:331–41. 10.1016/j.jns.2016.11.067 28017241

[47] Marrie RA Fisk JD Yu BN Leung S Elliott L Caetano P. Mental comorbidity and multiple sclerosis: validating administrative data to support population-based surveillance. BMC Neurol. (2013) 13:16. 10.1186/1471-2377-13-16 23388102 PMC3599013

[48] Thomas C Schneider BT Verza CS Fassina G Weber LR Moreira M. Prevalence of fibromyalgia in a Brazilian series of patients with multiple sclerosis. Arq Neuropsiquiatr. (2023) 81:803–8. 10.1055/s-0043-1772673 37793402 PMC10550347

[49] Téllez N Río J Tintoré M Nos C Galán I Montalban X. Fatigue in multiple sclerosis persists over time: a longitudinal study. J Neurol. (2006) 253:1466–70. 10.1007/s00415-006-0247-3 16773265

[50] Donzé C Massot C Defer G Vermersch P Lecoz P Derepeer O. NUTRISEP: Assessment of the nutritional status of patients with multiple sclerosis and link to fatigue. Rev Neurol (Paris). (2023) 179:282–8. 10.1016/j.neurol.2022.10.004 36792421

[51] Koch M Mostert J Heerings M Uyttenboogaart M De Keyser J. Fatigue, depression and disability accumulation in multiple sclerosis: a cross-sectional study. Eur J Neurol. (2009) 16:348–52. 10.1111/j.1468-1331.2008.02432.x 19490071

[52] Lenz ER Pugh LC Milligan RA Gift A Suppe F. The middle-range theory of unpleasant symptoms: an update. ANS Adv Nurs Sci. (1997) 19:14–27. 10.1097/00012272-199703000-00003 9055027

[53] Motl R W McAuley E. Symptom cluster as a predictor of physical activity in multiple sclerosis: preliminary evidence. J Pain Symptom Manage. (2009) 38:270–80. 10.1016/j.jpainsymman.2008.08.004 19329276

[54] Bakshi R Shaikh ZA Miletich RS Czarnecki D Dmochowski J Henschel K. Fatigue in multiple sclerosis and its relationship to depression and neurologic disability. Mult Scler. (2000) 6:181–5. 10.1177/135245850000600308 10871830

[55] Mustač F Pašić H Medić F Bjedov B Vujević L Alfirević M. Anxiety and depression as comorbidities of multiple sclerosis. Psychiatr Danub. (2021) 33(Suppl 4):480–5. 34718269

[56] Cadegiani FA Kater CE. Adrenal fatigue does not exist: a systematic review. BMC Endocr Disord. (2016) 16:48. 10.1186/s12902-016-0128-4 27557747 PMC4997656

